# Enhancement of Ambient Mass Spectrometry Imaging Data by Image Restoration

**DOI:** 10.3390/metabo13050669

**Published:** 2023-05-19

**Authors:** Yuchen Xiang, Martin Metodiev, Meiqi Wang, Boxuan Cao, Josephine Bunch, Zoltan Takats

**Affiliations:** 1 Department of Metabolism, Digestion and Reproduction, Faculty of Medicine, Imperial College London, London SW7 2AZ, UK; martin.metodiev@npl.co.uk (M.M.); m.wang21@imperial.ac.uk (M.W.); a.cao21@imperial.ac.uk (B.C.); 2 National Centre of Excellence in Mass Spectrometry Imaging (NiCE-MSI), National Physical Laboratory (NPL), Teddington TW11 0LW, UK; josephine.bunch@npl.co.uk

**Keywords:** ambient mass spectrometry imaging, image restoration, single-image super-resolution

## Abstract

Mass spectrometry imaging (MSI) has been a key driver of groundbreaking discoveries in a number of fields since its inception more than 50 years ago. Recently, MSI development trends have shifted towards ambient MSI (AMSI) as the removal of sample-preparation steps and the possibility of analysing biological specimens in their natural state have drawn the attention of multiple groups across the world. Nevertheless, the lack of spatial resolution has been cited as one of the main limitations of AMSI. While significant research effort has presented hardware solutions for improving the resolution, software solutions are often overlooked, although they can usually be applied in a cost-effective manner after image acquisition. In this vein, we present two computational methods that we have developed to directly enhance the image resolution post-acquisition. Robust and quantitative resolution improvement is demonstrated for 12 cases of openly accessible datasets across laboratories around the globe. Using the same universally applicable Fourier imaging model, we discuss the possibility of true super-resolution by software for future studies.

## 1. Introduction

Mass spectrometry imaging (MSI), has played an indispensable role in the field of metabolic imaging in the last decade. Since its inception in the last century [1], MSI now comprises a wide array of techniques that all grant the ability to map the distribution of biochemical species by taking spatially resolved mass spectra, i.e., hyperspectral imaging. In comparison to more traditional imaging modalities, MSI is especially attractive in its intrinsic chemical richness that can be attributed to thousands of *m/z* channels acquired in a parallel fashion in certain types of mass spectrometers, which can be subsequently identified to be molecules of interest, without the need for labelling. As such, MSI has found an impressive number of applications, including clinical diagnostics, drug metabolism and toxicology, oncology, etc., which have been reviewed in more depth elsewhere [2]. The most widely used MSI techniques are often cited to be matrix-assisted laser desorption ionisation (MALDI) and secondary ion mass spectrometry (SIMS). Their desirability stems from the excellent sensitivity and spatial resolution that can be achieved, as MALDI is fundamentally governed by the imaging optics assuming optimised matrix quality and SIMS only by the ionic spacing used in the primary beam and the guiding optics, which theoretically can be on the order of 10 nm [3].

There has been, however, significant effort in the development of *ambient* MSI (AMSI) methods which are capable of imaging samples in their native form without the need for sample preparation or high-vacuum conditions. With a clear advantage in terms of direct analysis, AMSI was first demonstrated [4] in the form of desorption electrospray ionisation (DESI) mass spectrometry, first introduced in 2004 [5]. Since then, AMSI has seen a multitude of applications in the analysis of animal, human, and plant tissues; microbes; and even forensic analysis [6,7,8,9], employing a variety of novel ionisation techniques. Despite promising results, AMSI is still limited by the lack of spatial resolution, which lies mostly within the 10 s–100 s μm range [2]. This is further exacerbated by the well-documented trade-off in hyperspectral imaging between spatial resolution and imaging speed, which is also a major hindrance for the wider applicability of MSI for clinical applications, where reasonably fast and cost-effective operation is needed. Furthermore, sacrificing imaging speed for resolution may be counterproductive, since slower runs also involve issues such as diminished sensitivity and the emergence of artefacts, both of which have been shown to decrease the effective resolution of the AMSI data [10]. A major concurrent research effort has focused on achieving superior spatial resolution. For instance, hardware solutions based on laser desorption have demonstrated notable improvement, bringing the sampling pixel size down to the ≤10 μm range [11]. Given the multiple different ionisation methods, each with different intrinsic limitations, the measure of spatial resolution itself has yet to be rigorously standardised and quantified as there is a lack of consensus in the field [12]. While the systematic comparison of different AMSI modalities and setups remains a challenge, the ongoing pursuit for higher resolution will likely reach the (sub)cellular level in the near future [13]. Such high resolution will be achieved, however, at the cost of imaging speed.

Complementary to hardware optimisation, software solutions can also be implemented post-acquisition with virtually zero cost. While some existing work have proposed the enhancement of effective contrast and resolution by means of image fusion [14], the majority of recent efforts have been devoted to implementing state-of-the-art algorithms to maximise the retrieval of information and its interpretability, in terms of e.g., ion identification [15]. To our knowledge, there has been no documented image restoration method employed that can be applied to AMSI data on a single-image level without any prior information. To fill this gap, here we propose and demonstrate the development and application of two fully-automated algorithms for the enhancement of ambient MS images. The first method builds on our previous work that has aimed to provide a modality-agnostic measure of resolution [16], which is used here to automate a Richardson–Lucy (RL)-type deconvolution routine that requires no other user input. The second pipeline demonstrates the application of deep learning (DL) networks to AMSI, starting from pre-trained weights that were trained on a set of natural images. The resulting trained weights are directly applied on unseen AMSI data to provide image denoising, artefact removal, as well as resolution up-scaling. The performance of our image enhancement methods are then demonstrated on 12 openly available datasets from Metaspace [17]. The results allow us to have a global image resolution and quality comparison across different groups and instrumental parameters, thus shedding light on some under-discussed subtleties and imaging characteristics of different ambient modalities.

## 2. Materials and Methods

### 2.1. Laser Desorption Rapid Evaporation Ionisation Mass Spectrometry (LD-REIMS) Imaging

LD-REIMS is a relatively new AMSI modality that provides an optically induced laser-based desorption process enhanced by collision surface post-ionisation, whose resolution can be controlled by the corresponding optics while retaining its preparation-free nature and sensitivity [18]. Mouse brain samples were imaged using an in-house setup consisting of a Opolette HE2731 (Opotek, Carlsbad, CA, USA) optical parametric oscillator (OPO) operated at 20 Hz and 2.94 μm tuned for mass spectrometry imaging and a distal optical system constructed of optomechanical components (Thorlabs Inc., Ely, UK) and an aspheric lens (C028TME-E, Thorlabs Inc., Newton, NJ, USA) to correct for aberrations. The beam diameter was measured to be approximately 30 μm at the sample plane, where microscope slides can be mounted on an in-house built stage (Thorlabs Inc., Newton, NJ, USA) and coupled to a Xevo G2-XS QTOF mass spectrometer (Waters Corporation, Milford, MA, USA) controlled by MassLynx 4.1 software (Waters Corporation, Milford, MA, USA). The mass spectrometer was equipped with a prototype REIMS source described by Jones et al. in detail [19]. For general imaging analysis, the acquisition rate was set to 2 scans/s with a stage speed of 100 μm/s and a pixel size of 100 μm. For high-resolution (10 μm pixel/s), a prototype optical parametric (OPA) system operating in the picosecond regime [20] was used in place of the OPO while keeping the remaining components identical. All mass spectrometric analysis was performed in the negative ion mode with a spectral range of 50–1200 *m/z*.

### 2.2. Data

The datasets utilised in this study are all openly available from Metaspace [17] and are summarised in Table 1. The choice of data has been determined to cover concurrent AMSI techniques, namely DESI data from Sheffield Hallam University (SHU), University of Copenhagen (U Copenhagen), University of Texas at Austin (UT Austin), and Imperial College London (ICL); laser-ablation electrospray ionisation (LAESI) and nanoDESI imaging data were obtained by the Pacific Northwest National Laboratory (PNNL); infrared-matrix-assisted laser desorption DESI (IR-MALDESI) data from North Carolina State University (NCSU); and LD-REIMS data were obtained in-house by our group at ICL.

### 2.3. Auto-Deconvolution by PSF Estimation

While the term deconvolution has generally been used in the field of mass spectrometry in the context of separation and extraction of spectral information [21], it is used here and in imaging theory in quite a different sense. Despite the aforementioned inconsistency in the quantification of spatial resolution, we have previously discussed the validity and applicability of a linear imaging model in MSI [16]:

(1) I=O⊗h+N

where the final image *I* can be treated as a convolution of the object *O* and a function *h* which is the so-called point spread function (PSF) of the MSI system, *N* is an additive noise term experienced by realistic systems. The PSF is also equivalently the image of a point object that has experienced the effective ‘blurring’ due to the imperfection of a finite imaging system. The PSF is hence usually taken as a measure for spatial resolution, and the deconvolution of the image by the PSF is referred to as deblurring. In the case of a frequently assumed Gaussian PSF, this same parameter has been used in MSI as the ‘86–14% criterion’ obtained from an edge intensity profile [22].

While extensive work exists for deconvolution in bioimaging in general [23], none to our knowledge has been applied to MSI. This is due in part to the two main challenges faced by any deconvolution problem—(1) the accurate estimate of an initial guess PSF, and (2) the termination criterion of the iterative algorithm used to deduce the PSF. Building on our previous work that provided an empirical method of estimating a 1D PSF (or line spread function), we have tackled both challenges by automating Richardson–Lucy blind deconvolution [24]—a commonly utilised Bayesian iterative method by means of object detection.

To automate the blind Richardson–Lucy deconvolution algorithm, both the initial guess and the termination criterion are facilitated by the evaluation of a 1D PSF with the method previously discussed in [16]. A flow chart of the algorithm can be found in Supplementary Materials. In summary, the line spread function (1D PSF) of a given image is estimated by first detecting the sharp edges by means of Canny detection and Hough transformation [25,26]. The intensity profiles perpendicular to the detected edges are then differentiated and Gaussian fitted to produce the corresponding PSFs. Successful fits ($R^{2}>0.5$) are then combined into an initial guess PSF to start the RL deconvolution by assuming a 2D Gaussian profile whose width (2σ) is the mean from all included fitting results, which is considered a direct measure of blur. To determine when the deconvolution needs to be terminated, the width of the 2D PSF for the deconvolved version of the image at each iteration is calculated again, and the algorithm stops if |Δ2σ|<0.01 or Δ2σ>0, which indicate that either the change in resolution is plateauing or having an adverse effect (increasing blur). To cater for stabilisation effect, a minimum of 5 iterations is set before the termination criterion is effective. Both the final image and estimated PSF are returned as outputs.

### 2.4. Training and Inference with GANUNET

#### 2.4.1. Model Architecture

The overall model architecture used in this work can be found in the Supplementary Materials. In summary, two different kinds of deep neural networks have been specifically trained, optimised, and used in conjunction for image restoration of AMSI data. The first is enhanced SRGAN (ESRGAN) [27], a type of generative adversarial network (GAN) [28]. GANs are based on an adversarial system between two separate neural networks: the generator and the discriminator. In the context of imaging, the generator network constructs ‘fake’ samples while the discriminator evaluates whether the fake samples generated by the generator are distinguishable from the ground truth images. The back-propagation allows the output of the discriminator to train the generator. The training purpose for the generator is to generate samples that fool the discriminator, which is also learning constantly. The learning process for the discriminator is to classify whether the input (the generated images) is real or fake according to the ground truth images. ESRGAN builds on its predecessor, SRGAN [29], a pioneering technique that can generate realistic upsampled textures based on a single-image input (i.e., single image super-resolution (SISR)) but suffers from unwanted hallucination artefacts. To overcome this limitation, ESRGAN introduced residual-in- residual dense blocks (RRDBs) as the basic building block, a probabilistic adversarial loss, and a perceptual loss that is more related to naturally perceived image quality.

While the trained weights from ESRGAN provide perceptually better upsampled images in general (Supplementary Materials), we observe that when applied to unseen data of different tissue types, they tend to suffer from the checkerboard artifact [30] as the one-to-many mapping requested from the upsampling process causes ambiguities. This artifact can potentially be remedied by training with a much larger amount and variety of training data as well as (re-)introducing normalisation blocks [30]. In the interest of a low-cost model and training routine that can be easily replicated across laboratories, we have trained a second network that serves as both a de-artifacting and denoising block. This network is based on the U-Net, a deep learning network with an encoder-decoder architecture [31]. While a similar network has been previously used for image restoration of fluorescence microscopy images [32], we note that neither the network depth nor the synthetic data training strategy are directly applicable to AMSI as high level of hallucination was observed. As such, we have trained a 5-level model that is referred to as ‘UNET5’ henceforth.

Both trained weights from ESRGAN and UNET5 can be sequentially applied to raw mass spectrometry images that have been pre-selected accordingly (Supplementary Materials) to carry out image restoration, hence giving the workflow its name of GANUNET.

#### 2.4.2. Model Training and Optimisation

Considering the small number of AMSI images compared to the vast numbers of natural images available for training, optimised weights for routine AMSI applications were obtained by transfer learning. The training process for ESRGAN consists of three main steps: (1) First, an SNR-oriented model with mean absolute error loss is trained on natural images, which is then used to initialise the generator. (2) The generator and discriminator are then trained with perceptual loss and adversarial loss until the network converges. (3) The weights of the pre-trained model are fine-tuned using the same strategy using mass spectrometry images generated from pork liver samples (3757 images), which are widely available at minimal cost, thereby providing spectral and spatial features typical of biological tissues. UNET5 was also trained with the same liver data used for training ESRGAN with a synthetic noise adding strategy. Details on the training strategy are also included in the Supplementary Materials document. The hyper-parameters used for training have been optimised empirically and summarised below in Table 2:

#### 2.4.3. Measuring Resolution with Fourier Ring Correlation

Fourier ring correlation (FRC) is another approach to measure the image resolution which has recently gained traction in fluorescence microscopy [33]. While MSI resolution has traditionally been measured using a PSF without the consideration of noise, recent works have recognised that noise can also limit spatial resolution [34,35,36]. FRC also computes an effective PSF while a noise level is also estimated adaptively. Therefore, while it is schematically similar to our approach [16] and also modality agnostic, in practice it is easier to implement and more robust as it removes the need of a step edge ROI. Fundamentally based on the same imaging model that we have adopted here, a cut-off frequency is computed in FRC beyond which there is insufficient details to be identified in the frequency domain due to noise to determine the image resolution. This cut-off frequency is selected based on a manually defined threshold, generally chosen with prior knowledge of the relative SNR. While FRC traditionally needed two images to be correlated against each other, we adopt a modified form of the single-image correlation method proposed by Koho et al. [33]. The selection of the most appropriate threshold nevertheless remains the most important task in producing accurate FRC. In the scope of information theory, we adopt the ‘1/2-bit’ threshold that is proposed by Van Heel et al. [37], which is robust across different types of images due to its direct correlation with the amount of information obtainable while retaining convergence even when the sampling is low (i.e., not many pixels in the images correlated). After obtaining the frequency cut-off ($f_{thresh}$), the corresponding resolution (*δx*) can be calculated by:

(2) $$\delta x=\frac{2}{f_{thresh}}\times \Delta x$$

where Δx is the pixel size used.

## 3. Results

### 3.1. Auto-Deconvolution of Ambient Mass Spectrometry Images

**Figure 1:**
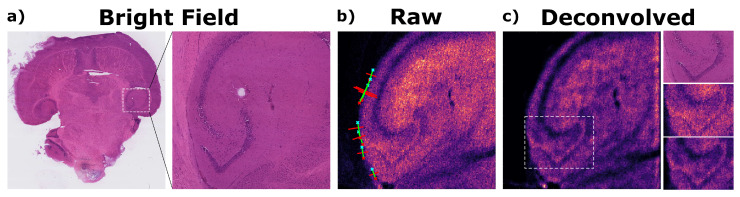
Demonstrating a region of interest from a mouse brain indicated in (**a**) the corresponding bright-field optical image, imaged in high-resolution by LD-REIMS; the effect of applying the auto-deconvolution algorithm is highlighted by comparing the (**b**) image of the highest intensity, tissue-specific ion (*m/z* = 600.5) before & (**c**) after 5 iterations of deblurring. A specific spatial feature is further zoomed-in on and compared with the same region from the optical image.

To demonstrate the effectiveness of our approach, non-background peaks from Dataset 11 and their corresponding ion images have been deconvolved using our algorithm, as described in Section 2.3. An example of the results is presented in Figure 1.

By inspection, effective denoising is achieved and some features become visibly more pronounced, as confirmed in the corresponding optical image. To quantify this deblurring effect, the resolution of the image before and after the iterative deconvolution process can be evaluated using our PSF estimation method along naturally ‘sharp’ edges in the field of view, which is automated by edge detection algorithms (Section 2.3) and visualised as green lines in Figure 1. As a result, the width (2σ) of the estimated PSFs indicate that the resolution has indeed improved by about 40% as it decreased from 2σ=3.63 pixels to 2σ=2.15 pixels.

**Figure 2:**
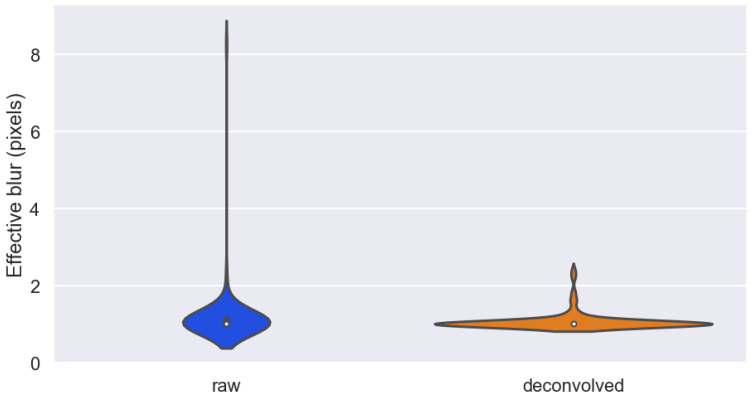
A violin plot that approximates the distributions of effective blur (2σ) in the top 100 tissue images from Dataset 11 in its raw form and after the application of auto-deconvolution. Clear outliers have been removed before visualisation.

By repeating the same process for all 102 images, a more statistically relevant comparison can be made. Figure 2 shows the relative distributions of PSF width before and after deconvolution. It can be seen that the effective blur in the images have generally been restored to approach the information theoretic limit of a single pixel with the aid of deconvolution, as the waist of the distribution settles around 1. To confirm this observation statistically, a single-sided Wilcoxon signed-rank test has been performed on the obtained resolution parameters, which produced a test statistics of 1860 and a corresponding *p*-value of 9.7 × 10${}^{-3}$, which allows the rejection of the null hypothesis that the deconvolution algorithm has no effect on the blurring parameters.

While it is possible to perform deconvolution on every image in any MSI dataset, the time needed per image varies radically as it largely scales with the initial estimation of the PSF and hence the number of iterations needed to reach the pre-defined termination criterion. To give some context, this ranges from 2 s to 22.5 s for the images experimented on in this case with a standard workstation. As MSI datasets can easily comprise 10^3^–10^4^ images, dataset-level deconvolution should only be attempted with the consideration of computational intensity. Additionally, not every image can be adequately deconvolved—some subtle considerations and some general rules in selecting the deconvolvable images are discussed in the next section.

### 3.2. Image Restoration by GANUNET

**Figure 3:**
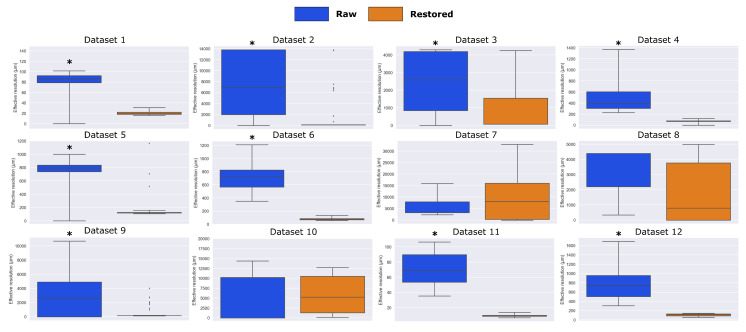
Comparison of the FRC-calculated spatial resolutions of 12 AMSI datasets before & after the application of GANUNET. The datasets are obtained from different laboratories across the globe with varying modalities and parameters, specified in Section 2.2. Datasets where a statistically significant improvement in resolution has been observed are marked by an asterisk.

By using the trained weights obtained via the strategy outlined in Section 2.4, all 12 open-access AMSI datasets have been upsampled (4X on both axes) and enhanced. To study the effective resolution change achieved, FRC analysis [33] adapted for AMSI has been used to investigate the noise-limited cut-off in the spatial frequency domain, which also allows the calculation of an effective PSF and hence resolution. The resolutions calculated for the 12 datasets are presented in Figure 3. It is evident that a clear improvement in resolution is observed across majority of the cases, where the rejection of the no-change null hypothesis have been statistically tested (one-sided Wilcoxon) and marked by an asterisk where applicable. No significant improvement was observed for the nanoDESI datasets (7 and 8). As the resolution improvement measured by FRC predominantly comes from a denoising effect, the outcome depends on the intrinsic noise level of the data. Interestingly, Datasets 7 and 8 registered the lowest images SNRs overall (Supplementary Materials), which most likely led to the limited performance of GANUNET in this case. It should also be noted that the performance of both the FRC metric and GANUNET will suffer from low number of pixels, i.e., under-sampling, which is discussed in more detail below. For example, Dataset 10 has pixel dimensions of 35 × 70, meaning that the FRC algorithm is estimating resolution using two sub-sampled images (∼17 × 35 pixels) derived from the original image, limiting the accuracy and hence comparability with those from larger images.

**Figure 4:**
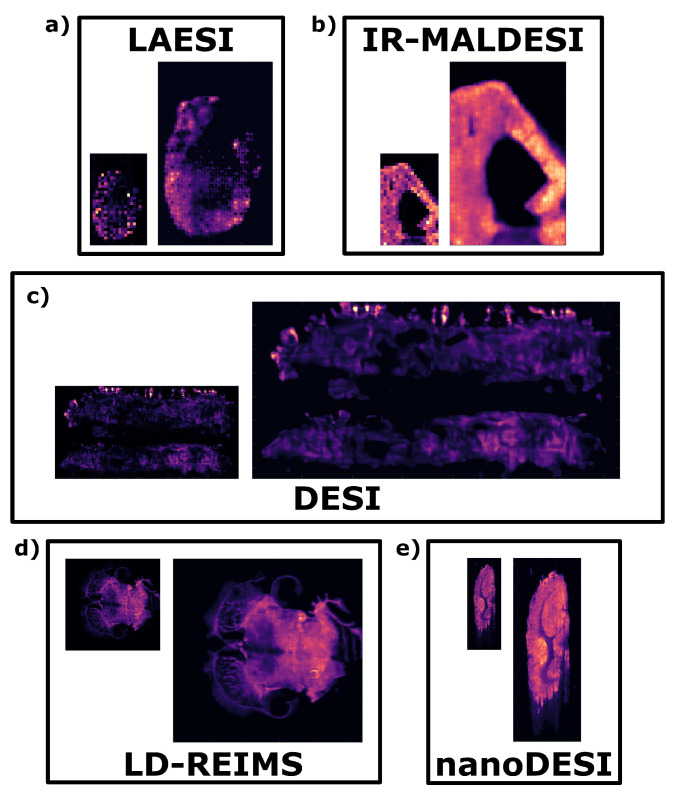
Selected ion image comparisons to demonstrate the effect of GANUNET; both the raw and GANUNET-restored and upscaled images are displayed in pairs, and a 2-fold difference in size is adopted for easier visual comparison while retaining the aspect ratios. The ion images with the highest spectral intensity and discernible sample-specific features have been chosen to represent different modalities and sample types. Specifically, (**a**) (*m/z* = 806.57) a mouse kidney imaged by LAESI (Dataset 9), (**b**) (*m/z* = 255.23) a rat liver section imaged by IR-MALDESI (Dataset 5), (**c**) (*m/z* = 617.50) sections of a whole Galleria mellonella imaged by DESI (Dataset 2), (**d**) (*m/z* = 862.65) a mouse brain section imaged by LD-REIMS (Dataset 11), and (**e**) (*m/z* = 820.52) a rat brain section imaged by nanoDESI (Dataset 7).

To visually illustrate the generalisability of the trained GANUNET models, selected ion images are shown in Figure 4. Images have been chosen from five different datasets so as to represent the five different AMSI modalities covered in the study.

**Figure 5:**
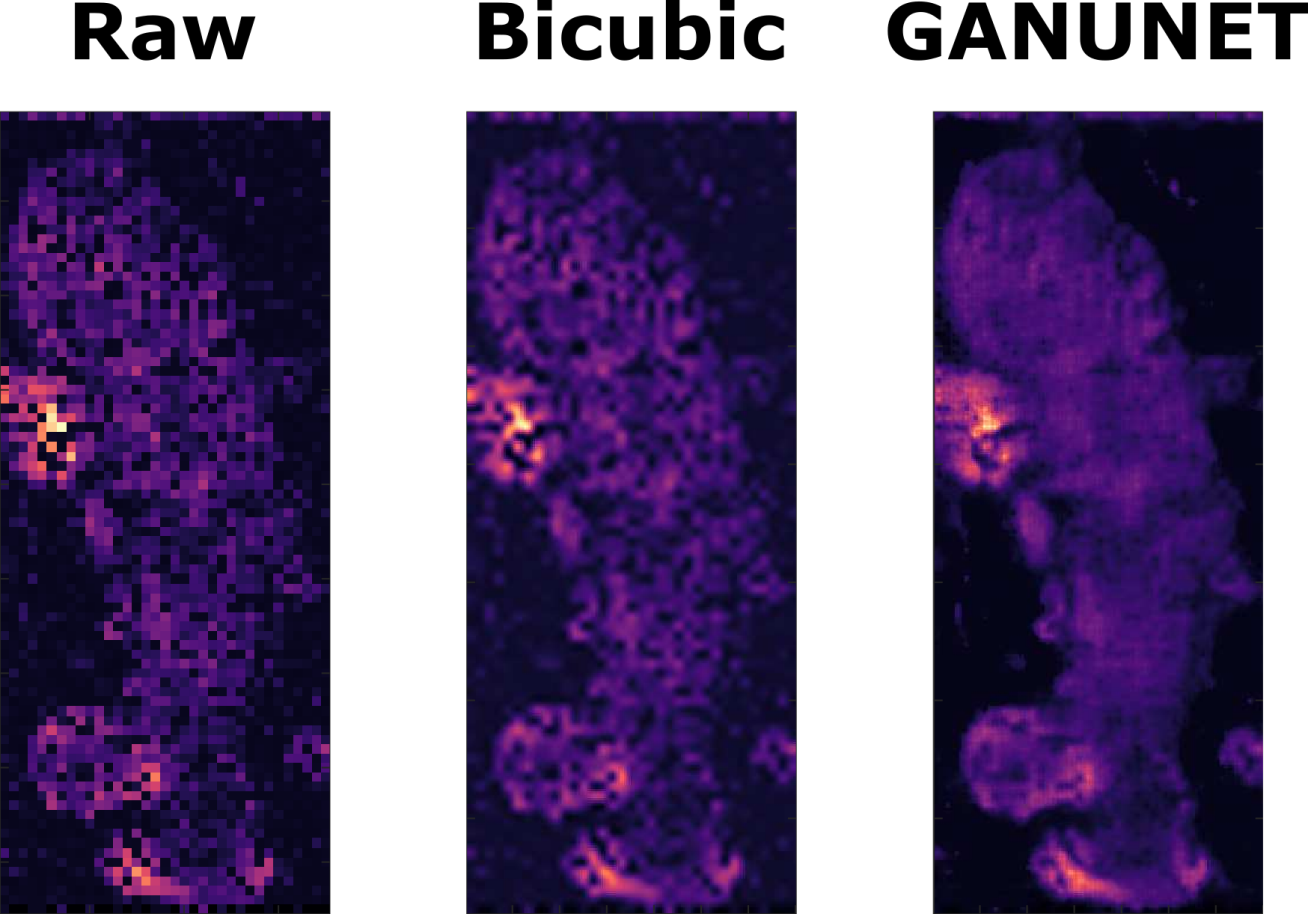
An example ion image (*m/z* = 213.87) from Dataset 3 where the raw data (LEFT) suffers from low SNR and hence missing values. The results of upsampling operations on the same image from standard bicubic interpolation (CENTRE) and GANUNET (RIGHT) are also compared.

In addition to the perceptually observable denoising effect, the four-fold upsampling effect of GANUNET also interpolates intensity values based on the machine learned understanding gained from the liver samples used in training. Notably, the performance of this inference is palpable when there is a high level of missing or incomplete information in the raw data, which is no uncommon in AMSI datasets due to the low detectability of certain ions, which may be of biological interest. Figure 5 presents such an example from Dataset 3, where GANUNET successfully fills the ‘missing pixels’ in the raw data without observable hallucination or artifact. This is then further compared to the 4X upsampled image obtained from standard bicubic interpolation, which is seen to have little effect on the overall quality of the image as well as inducing some blur.

**Figure 6:**
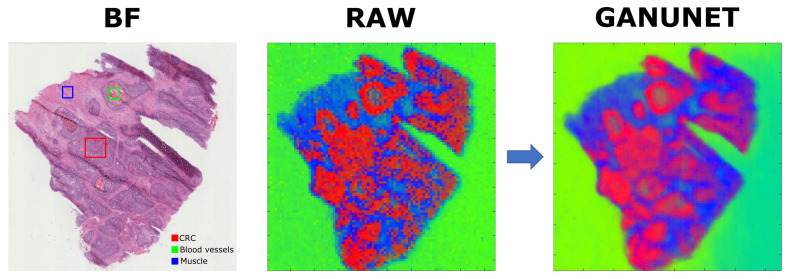
The bright-field (BF) optical image with staining is compared to false-colour images composed of the mapping of scores for the first three principal components obtained from Dataset 4 before & after applying GANUNET. The associated pathological tissue features [38] have also been indicated in representative regions in the BF image, namely red = colorectal cancer (CRC), green = blood vessels, blue = muscle.

Additionally, to demonstrate the effect of GANUNET on common downstream analysis workflows that are widely used in MSI, image segmentation by means of principal component analysis (PCA) has been performed on Dataset 4 after applying variance stabilising transforms [38]. The relative abundance for the first three PCs of the analysis are then reshaped and combined into false-colour images for visual comparison. The results are found in Figure 6, where once again discernible denoising and sharper distinction of spatial features are evident. When compared to the histological image of the same sample, it can be seen that certain pathological features that were previously distorted by noise and artifact have been restored. In conjunction with the 16-fold increase in total number of pixels available, subsequent spatial investigation by, e.g., correlating selected region(s) of interest with pathological annotations should become considerably simpler and more accurate. To this end, GANUNET is an important addition to the emerging computational tools and their wider adoption in terms of multimodal integration and correlative imaging, highlighted to be a concurrent trend in MSI [10]. The alignment of optical and other imaging data in general, e.g., MRI can be facilitated by the simple implementation of GANUNET as a single pre-processing step. The precise overlap of multi-modalities can then be further exploited to potentially enable multi-omic analysis on a single-cell level. Similar to the approach proposed by Rappez et al. [39], GANUNET can in principle bypass some hardware limitations of AMSI and facilitate the spatial investigation of new and even existing datasets at a cellular level.

**Figure 7:**
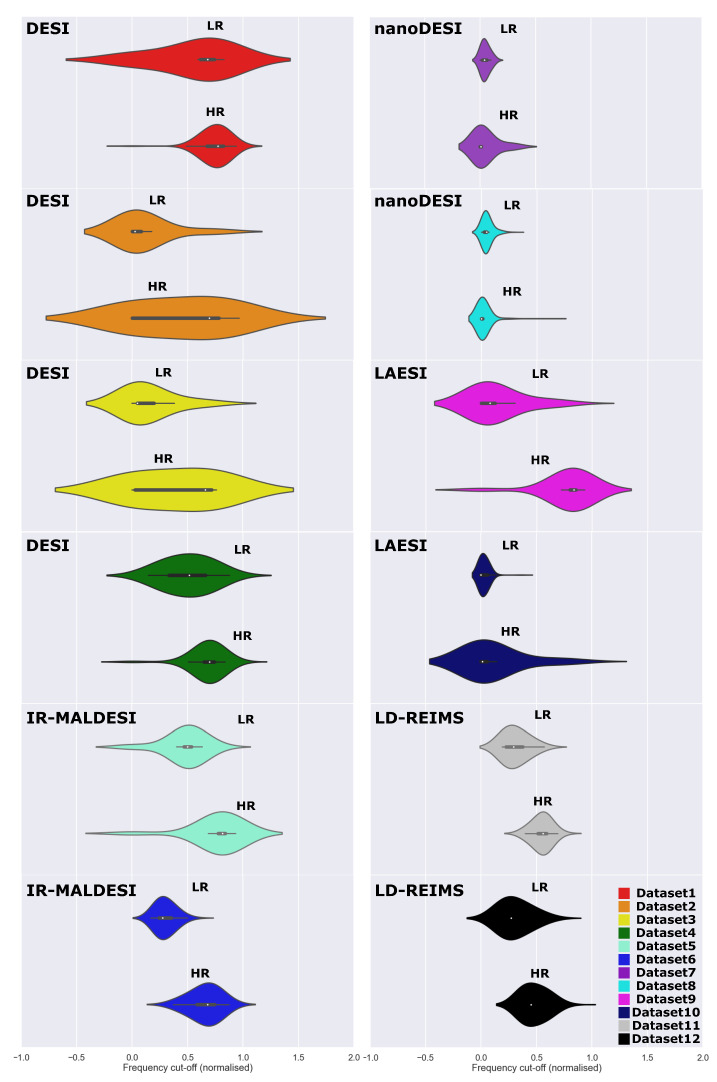
Violin plots of the cut-off frequencies deduced by FRC for each dataset with and without restoration by GANUNET. The relative distribution of each bin is calculated with a fixed kernel bandwidth of 1.0 and scaled by the number of observations in that bin. The results are always presented in pair, where LR refers to the ‘low-resolution’ raw data and HR refers to the ‘high-resolution’ restored images.

While the pan-modality comparison of resolution is not particularly meaningful due to the wide difference in the choice of sampling pixel size, the GANUNET workflow does allow us to evaluate the imaging performance and characteristics in terms of the cut-off spatial frequency, which is normalised and modality agnostic. As such, the comparison of frequency content for all 12 datasets is plotted in Figure 7. By inspection, both the effect of image restoration and dataset-specific characteristics may be visualised this way. The kernel density, for example, correlates to the maximum spatial frequencies detectable across the dataset, and it can be seen from Figure 7 that an elongation and/or shift of which is generally observed after restoration by GANUNET, suggesting that a shift towards higher spatial frequencies and hence better resolution. It is also interesting to note that the distributions are representative of the imaging characteristics. For instance, the LD-REIMS datasets and indeed other laser desorption-based modalities see a narrower spread in their spatial frequencies in general compared to DESI. This large spread towards low frequency cut-offs is intuitively in line when considering the nature of signal acquisition of DESI, which relies on a ‘pool’ of ionised analytes at the sample surface, which may spread to a different extent depending on their respective solubilities and may equate to a large blurring effect (wide PSF). This in turn depending on the relative intensity level of the image could translate to poor resolution. Similarly, the nanoDESI datasets studied here generally suffer from low frequency cut-offs/resolutions and are not significantly improved with GANUNET. Lastly, it is worth noting that both negative and frequency cut-offs >1 predicted by the kernel estimates are physically improbable and most likely due to outlying values in the datasets. The former is not possible within the scope of the Fourier imaging model used here, and the latter suggests ‘true’ super-resolution surpassing the limitations imposed by the hardware, which is not currently observed by us but is nonetheless a promising future direction.

## 4. Discussion

### 4.1. What Is Deconvolvable?

We have presented an automated blind deconvolution algorithm that was readily applied to AMSI data and gave statistically better overall resolution as a result. As with any good deconvolution algorithm, the key to the successful deblurring lies with the accuracy of the estimated PSF produced by the algorithm, which is affected by a number of factors. Firstly, the starting position of the estimate is critical, a grossly over-/under-estimated PSF would likely lead to poor deconvolution results, which is in this case governed by the accuracy of our initial PSF estimate. In practice, this translates to the ability to find clearly defined edges in the image and thereby obtaining a trustworthy Gaussian fit. By using the goodness of fit of the latter as a thresholding metric, we have noticed that the rejected images are generally noisy by inspection, thus placing an effective SNR requirement on the images that can be successfully deconvolved. Furthermore, while the Richardson–Lucy algorithm accounts for some Poisson noise (Supplementary Materials), any other noise sources would require better understanding of the noise model and refinement to be made specific to each AMSI modality.

**Figure 8:**
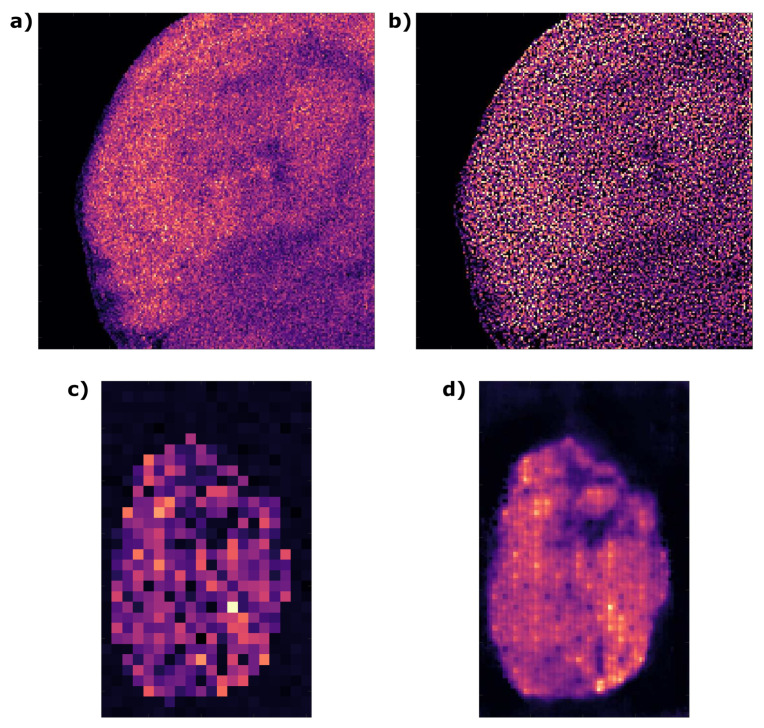
Application cases where the use of auto-deconvolution and GANUNET need careful consideration. (**a**,**b**) A case of noise amplification due to over-deconvolution from Dataset 11 where the spatial content in the ion image is replaced with noise. (**c**,**d**) Appearance of checkerboard artifact when a raw ion image from Dataset 9 is under-sampled.

Secondly, as the same estimate is used as a performance metric over iterations, spurious fitting results due to noise or otherwise can also lead to poor performance, causing, e.g., noise to take over due to too many iterations (Figure 8). Even if the initial guess is accurately fitted, it should be noted the fundamental assumptions of the PSF also play an role. Specifically, the 2D PSF is approximated in this case by assuming a rotational symmetry and is presumed to be isotropic across the image (i.e., the same for every pixel). Neither are necessarily true due to adverse imaging effect such as (asymmetric) aberrations [40] or a naturally asymmetric sampling beam. It would thus be interesting to implement 2D estimation methods in the future, by incorporating the directional information of edges [41] or indeed by combining with the FRC tool that we have already developed. Ultimately, one would need to make an informed decision on whether the image contains sufficient information for the resolution measures to be accurate.

Finally, we note that deconvolution is still fundamentally limited by the imaging hardware, which is to say that Shannon’s sampling theorem [42] should still be obeyed. As such, there are cases where the estimated PSF for an image would have a calculated width ≤1 pixel and thus cannot be used to perform further deblurring as that would indicate the extraction of sub-pixel detail without additional prior information and fundamentally violates the rules of information theory.

### 4.2. Success & Limitation of GANUNET

Within the 12 datasets that GANUNET has been tested on, the performance has been demonstrated to be generally robust both qualitatively and quantitatively. Considering that the models have been constructed with<4000 images and at minimal material cost, the utility of GANUNET as a single-step image restoration tool is highly attractive. While we believe that it can be generalised to wider use of most types of imaging sample and modality, it is of course not without limitation. Notably, the sampling employed during image acquisition is of vital importance and perhaps somewhat under-explored in AMSI. In imaging in general, it is considered good practice to use a sampling pixel size that is ≤smallest feature size that is desirable to resolve, e.g., a single cell. Often in MSI experiments, however, the pixel size is kept at a maximum to maintain the throughput. In this scenario, if the original data is *under-sampled* then GANUNET would have minimal or adverse effect, as illustrated by Figure 8c,d. With closer inspection, it can be seen the checkerboard artifact re-emerges even with the suppression from UNET5. Sufficient sampling (commonly referred to as ‘over-sampling’ in MSI terms) is thus an essential preamble for high-performance restoration. Within the same information theoretic framework discussed above, it also states that further resolution improvement is unlikely if it is ‘pixel-limited’. As such, while GANUNET can be considered a SISR algorithm at its core, we would regard it a highly optimised denoising and interpolation tool and do not expect GANUNET to be able to achieve ‘true’ super-resolution where novel features can be restored from under-sampled images. This limitation can theoretically be remedied with the re-training with additional high-quality data or architectural changes [30]. As such, while GANUNET achieves robust image enhancement with machine learned perception between high and low frequency contents in the PSF, just as in auto-deconvolution, its parameter space is much larger meaning more complex true optimisation but also much superior tuneability to suit use cases. At the same time, as the results have demonstrated even the ‘base’ model of GANUNET is generally capable of serving as a ‘one-stop’ solution that provides practical image restoration functions, such as upscaling, making it an easily implementable computational tool for MSI laboratories with minimal cost and specialist knowledge.

In addition to being a restoration tool, GANUNET also presents a unique opportunity in exploring the inter-laboratory comparison of resolutions and imaging characteristics with the implementation of the FRC metric. To this end, data mining of experimental metadata ranging from ionisation source, polarity, pixel size, etc., may lead to important discoveries that will further aid the standardisation and applicability of AMSI techniques in general. As the FRC metric is also intrinsically linked to the linear imaging model we have discussed, it would be a natural next step to also incorporate the deconvolution function into our DL algorithm. The real interest thus lies with the possibility of learning a physics-based model that ‘understands’ the true distinction of high and low-resolution with the knowledge of the PSF, thus holds the key to true, hardware-limitation-defying super-resolution.

## 5. Conclusions

We have presented two image restoration algorithms that for the first time provide quantifiable resolution enhancement to ambient mass spectrometry images via sofware-only means. Both methods function on the basis of a linear imaging model that can be considered universally in Fourier space, thus also granting us the ability to compare the imaging performance of different modalities and experiments, across laboratories. Both the deconvolution and DL-based tools are thus widely deployable as a single-step, open-access resource to enhance the quality of the increasingly popular AMSI data. While the current DL networks have been trained with the focus of robustness and low computational cost, we expect the modification towards a machine-learned physics-based model of this kind would lead to software-based resolution breakthroughs in the near future.

## Figures and Tables

**Table 1:** Summary of datasets used.

Dataset	Origin	Sample Type	Ionisation Source	Ionisation Polarity	Pixel Size
1	SHU	human lung	DESI	negative	30 μm
2	U Copenhagen	Galleria mellonella	DESI	positive	150 μm
3	UT Austin	human endometriosis tissue	DESI	negative	100 μm
4	ICL	human colon	DESI	negative	100 μm
5	NCSU	rat liver	IR-MALDESI	positive	200 μm
6	NCSU	mouse pancreas	IR-MALDESI	negative	100 μm
7	PNNL	rat brain	nanoDESI	positive	150 μm
8	PNNL	human kidney	nanoDESI	positive	50 μm
9	PNNL	mouse kidney	LAESI	positive	250 μm
10	PNNL	plant leaf	LAESI	positive	300 μm
11	ICL	mouse brain	LD-REIMS	negative	100 μm
12	ICL	mouse brain	LD-REIMS	negative	10 μm

**Table 2:** Summary of optimised hyper-parameters.

Network	Loss	Optimiser	Learning Rate	Patch Size	Epochs
ESRGAN	Mean Absolute Error	Adam	0.0004	(64,128)	100/100
UNET5	Mean Absolute Error	Adam	0.0004	(64,128)	30

## Data Availability

All data used in this study are openly accessible via Metaspace (https://metaspace2020.eu, last accessed 1 May 2023). Some image data that support the findings of this study are available in the corresponding repository at https://github.com/oycxyd/MSImgRes. All image processing, restoration and visualisation tools used in this work are open-source and available in the Github repository.

## Supplementary Materials

The following supporting information can be downloaded at: https://www.mdpi.com/article/10.3390/metabo13050669/s1, Figure S1: A flow diagram outlining automated blind deconvolution algorithm developed for this study; Figure S2: A flow diagram outlining the implementation of UNET5; Figure S3: Example images to illustrate the effect of different noise sources on MSI data; Figure S4: Architecture of ESRGAN adopted in this study; Figure S5: A flow diagram outlining the implementation of ESRGAN; Table S1: Other metadata of datasets used [43,44,45].

## Abbreviations

The following abbreviations are used in this manuscript:

AMSI	Ambient Mass Spectrometry Imaging
CNN	Convolutional Neural Network
DESI	Desorption ElectroSpray Ionisation
DL	Deep Learning
(E)SRGAN	(Enhanced) Super Resolution Generative Adversarial Network
FRC	Fourier Ring Correlation
GAN	Generative Adversarial Network
IR-MALDESI	InfraRed Matrix-Assisted Desorption ElectroSpray Ionisation
LAESI	Laser Ablation ElectroSpray Ionisation
LD-REIMS	Laser Desorption Rapid Evaporation Ionisation Mass Spectrometry
MALDI	Matrix-Assisted Laser Desorption Ionisation
MSI	Mass Spectrometry Imaging
OPA	Optical Parametric Amplifier
OPO	Optical Parametric Oscillator
PCA	Principal Component Analysis
PSF	Point Spread Function
RL	Richardson–Lucy
RRDB	Residual-in-Residual Dense Block
SIMS	Secondary Ionisation Mass Spectrometry
SNR	Signal-to-Noise Ratio
