# Supplementary material for: Enhancement of Ambient Mass Spectrometry Imaging Data by Image Restoration
Source: Metabolites. 2023 May 19;13(5):669. doi: 10.3390/metabo13050669 (PMC10222327; doi:10.3390/metabo13050669)
Supplement: Supplementary file 1 [file metabolites-13-00669-s001.zip › metabolites-2334059-supplementary.pdf]

Supplementary Materials

Auto-deconvolution algorithm workflow

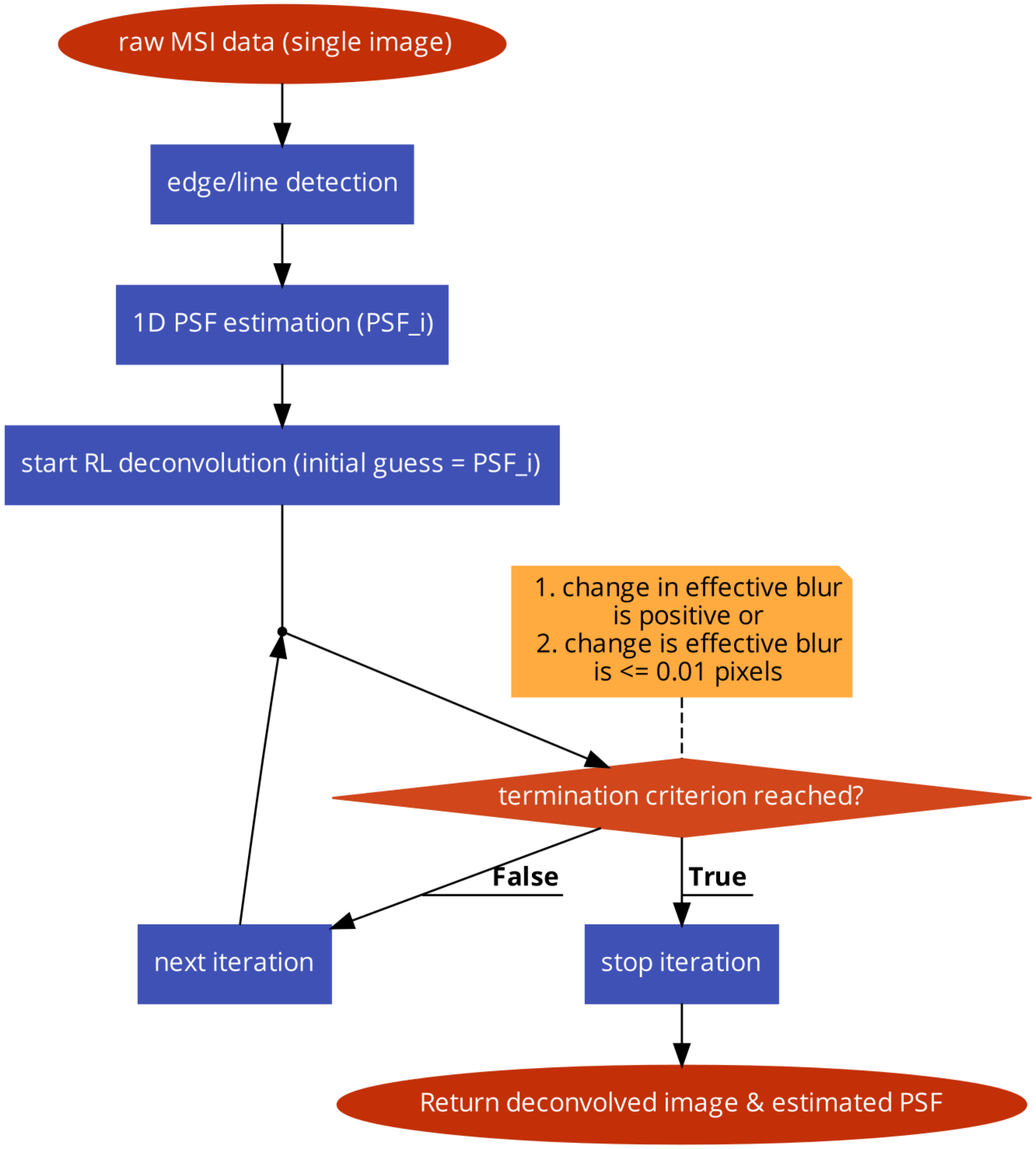

Figure S1. A flow diagram outlining automated blind deconvolution algorithm developed for this study.

Image pre-processing

From the raw data (.imzml format), each spectrum is transformed into an 8-level CWT domain where the biologically relevant peaks are manifested as ridges and detected using a stimulated annealing approach similar to that reported in [1], the parameters have already been fine-tuned for MSI data. As a result, the raw data matrix is reduced into lists of peaks detected. The remaining peak lists are then linearly interpolated to a common axis using the list with the most detected peaks as a reference. To facilitate deep learning and a fair comparison between datasets, further refinement of the pre-processed data was carried out by computing a weighted average of Structural Similarity Index (SSIM) and multi-SSIM [2] for images of every spectral feature with a representative reference image (in this case the total-ion-current image); those that produced a weighted average below a perceptually defined threshold were deemed to be belonging to either background or isotopes and hence removed. The remaining images are saved to .png format for training and/or image restoration.

Training UNET5 for denoising using synthetic noise adding[3]

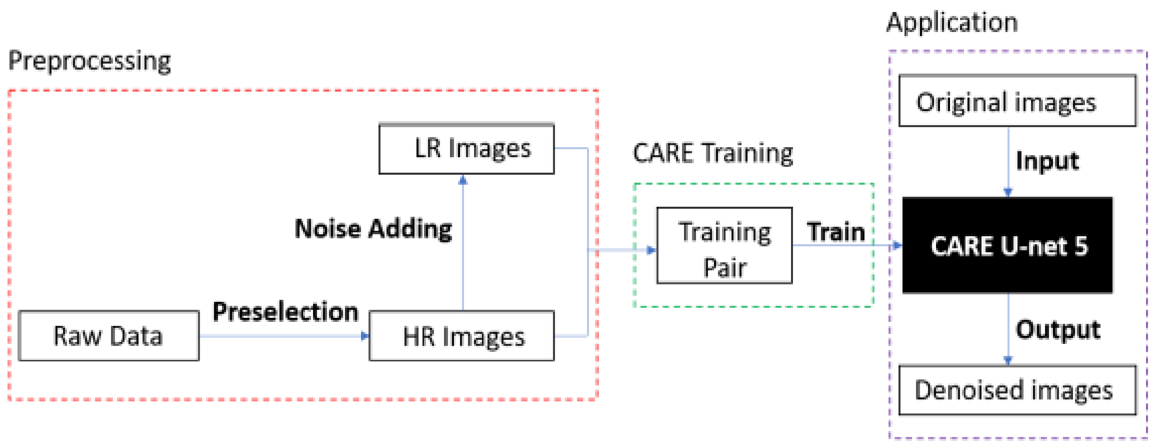

Figure S2. A flow diagram outlining the implementation of UNET5.

After removing background & noisy images using the procedure described above, the training datasets for UNET5 is formed by paring corresponding Low Resolution (LR) and High Resolution (HR) images. For denoising, LR counterparts are synthetically acquired by adding the appropriate type & amount of noise to the HR images. To optimise denoising performance, a realistic noise model of MSI is desirable. In this project, we have empirically optimised the synthetic data by assuming a linear detection model:

$$\text{Detected Signal} = \text{True Signal} + \text{Noise}$$

Which assumes an additive noise, that can be generally categorized as Gaussian noise and Poisson noise in distribution. Gaussian noise, also called white noise, is unrelated to pixel intensities of the original image, and it can be directly add to any given image. Poisson noise, or shot noise, however, is proportional to the intensity of each pixel. As the accurate noise source of (ambient) MSI systems has yet to be characterised, a combined noise model is proposed here, which balances both Gaussian noise and Poisson noise. An example of noise adding is presented in Fig. S4. The effect of Poisson

noise is by inspection relatively hard to observe visually compared to Gaussian noise, but it generally makes the image unsmooth and more resemblant of real MSI data. The noise level of Gaussian noise is determined based on a dataset-specific SNR, while the noise level of Poisson noise varies automatically with the pixel intensities of each image. As a result, patches from 3757 images (64 x 128 pixels in size, optimised in consideration of the MSI image sizes) with their noise-added counterparts were used to train the UNET5 weights utilised in this study. The hyperparameters were independently optimised and reported in the main text. The training time for UNET5 with 3757 images and 30 epochs only takes around 92 minutes.

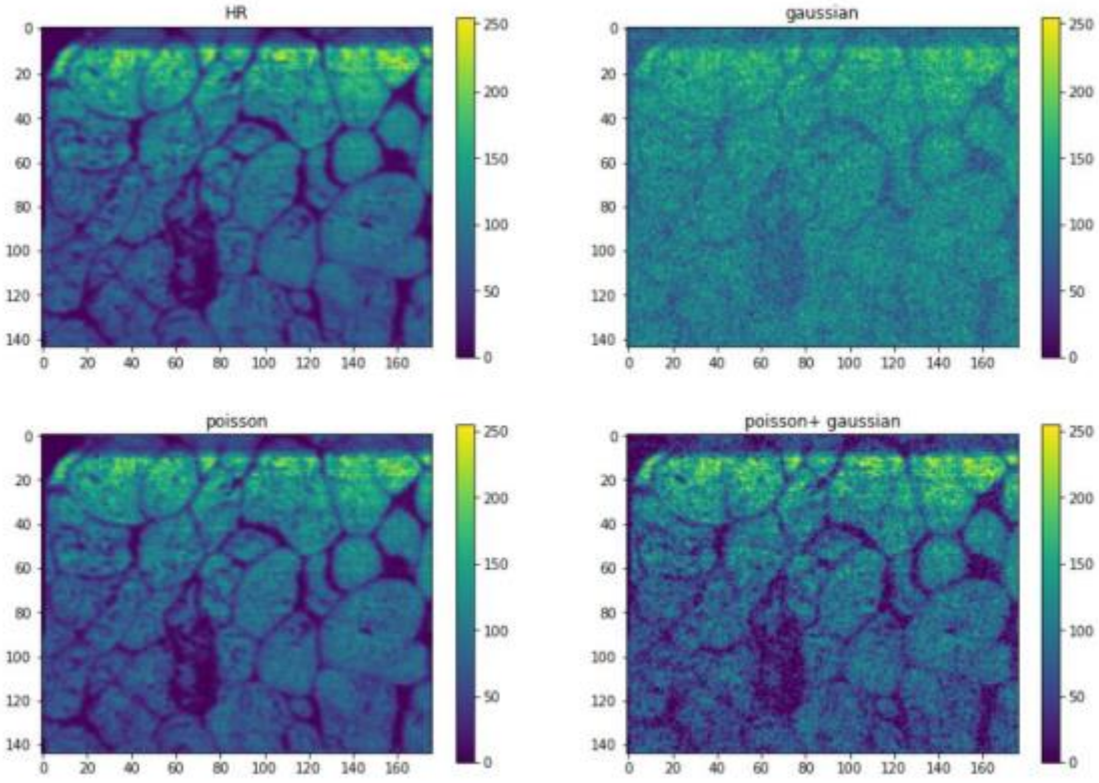

Figure S3. Example images to illustrate the effect of different noise sources on MSI data.

Training ESRGAN using synthetically generated LR-HR patches[3]

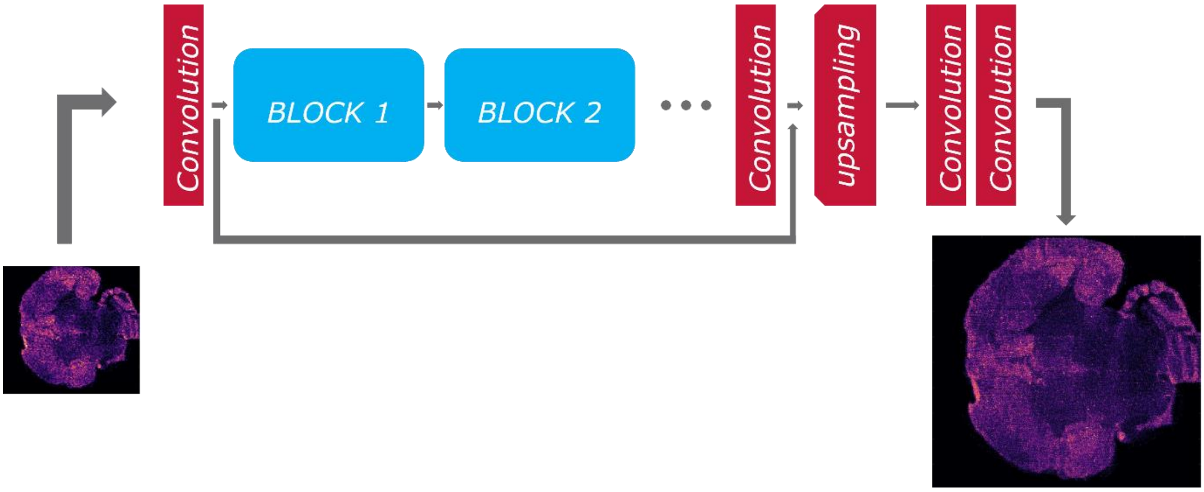

Figure S4. Architecture of ESRGAN adopted in this study.

The ground-truth or HR images for upsampling from the raw data went through the same preselection procedure as described above. LR counterparts are then acquired from downsampling the HR images by a factor of 4 using the bicubic interpolation. The training data for ESRGAN require 3-channel RGB images, however, each MSI ion image used in our image restoration is considered to be a single channel gray image. It was experimentally determined that using different combinations of ion channels to compose the RGB training image has little effect on the results. Therefore, we stack the same ion image 3 times to produce a fake RGB image for every training image. The training process started with pre-training a PSNR-oriented model with MAE loss. The generator was initialized by the pre-trained model. The generator and discriminator were then trained with perceptual loss and adversarial loss until it converged. The ratio of different losses was tuned during training, and it strongly affects the results. 100 epochs were used for pre-training and another 100 epochs for training the generator and discriminator network. The training time for ESRGAN for epochs takes around 6 hours, while 100 epochs of pre-training for initializing the generator also takes approximately 6 hours.

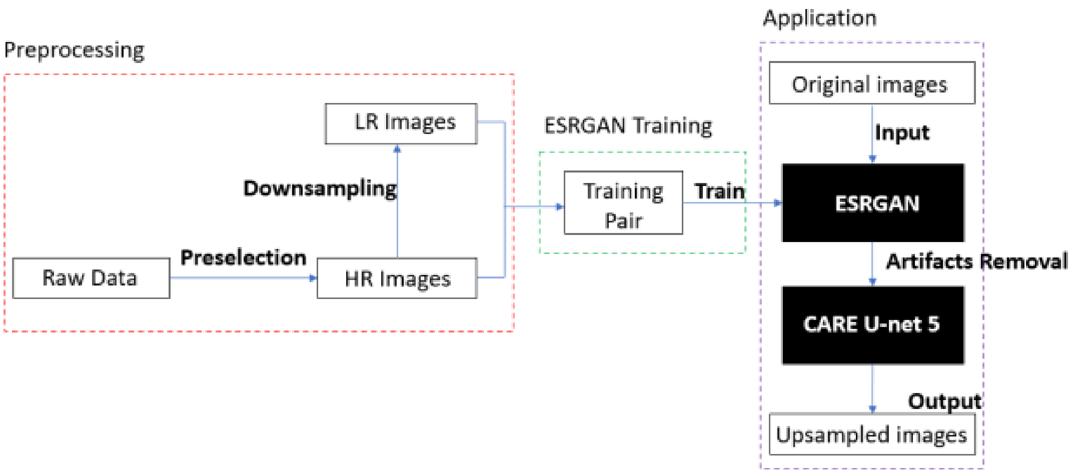

Figure S5. A flow diagram outlining the implementation of ESRGAN.

Other metadata of datasets used

The SNR of images in this study is calculated by:

$$SNR = 10\log_{10} < \frac{Img_i}{Img_n} >$$

where each dataset contains i number of images, each denoted by Img_i. A noise image Img_n is first determined for each dataset. This is nominally achieved by the taking the ion image with the lowest mean spectral intensity. In cases where strong background is observed, the noise image is selected by correlating every image with a reference image (e.g. the TIC image) that contains distinguishable spatial features of the sample, the structural similarity (SSIM) is then computed to be used as a metric. The image with the lowest SSIM value (i.e. the least similar) is deemed to be Img_n. Note that this measure is skewed in datasets that contain a high number of images with non-negligible background, thus the maximum SNR values are also included as well as the mean and standard deviation (st.d) to give a more complete picture.

Dataset	Pixel dimension	SNR (mean)	SNR (st.d)	SNR (max)
1	400 x 649	59.4498	8.647	71.0189
2	93 x 183	5.4031	5.8236	21.701
3	35 x 85	6.3565	4.6098	14.9555
4	94 x 94	32.3996	12.1707	46.231
5	19 x 30	6.1142	3.5375	26.3488
6	52 x 58	51.3555	6.6574	67.6074
7	79 x 214	3.6733	2.9763	15.2144
8	61 x 177	5.8911	4.4714	22.1777
9	20 x 32	31.0097	4.1681	53.5166
10	35 x 70	48.0376	8.1639	75.5312
11	133 x 129	29.7064	4.8345	49.3889
12	186 x 186	20.7867	3.3009	42.725

Table S1

References

[1] 10.1016/j.ijms.2016.09.020

[2] 10.1109/acssc.2003.1292216

[3] Deep Learning-assisted Enhancement for Mass Spectrometry Hyperspectral Images, Andy Cao, MRes thesis, 2022
